# Diaphragm dysfunction as a potential determinant of dyspnea on exertion in patients 1 year after COVID-19-related ARDS

**DOI:** 10.1186/s12931-022-02100-y

**Published:** 2022-07-15

**Authors:** Jens Spiesshoefer, Janina Friedrich, Binaya Regmi, Jonathan Geppert, Benedikt Jörn, Alexander Kersten, Alberto Giannoni, Matthias Boentert, Gernot Marx, Nikolaus Marx, Ayham Daher, Michael Dreher

**Affiliations:** 1 Department of Pneumology and Intensive Care Medicine, University Hospital RWTH Aachen, Aachen, Germany; 2 Institute of Life Sciences, Scuola Superiore Sant’Anna, Pisa, Italy; 3 Department of Cardiology, Vascular and Intensive Care Medicine, University Hospital RWTH Aachen, Aachen, Germany; 4 Department of Neurology with Institute for Translational Neurology, University Hospital of Muenster, Muenster, Germany; 5 Department of Medicine, UKM Marienhospital Steinfurt, Steinfurt, Germany; 6 Department of Intensive Care and Intermediate Care, University Hospital Rheinisch Westfaelische Technische Hochschule Aachen, Aachen, Germany

**Keywords:** Coronavirus, Mechanical ventilation, Long COVID, Diaphragm function, Dyspnea

## Abstract

Some COVID-19 patients experience dyspnea without objective impairment of pulmonary or cardiac function. This study determined diaphragm function and its central voluntary activation as a potential correlate with exertional dyspnea after COVID-19 acute respiratory distress syndrome (ARDS) in ten patients and matched controls. One year post discharge, both pulmonary function tests and echocardiography were normal. However, six patients with persisting dyspnea on exertion showed impaired volitional diaphragm function and control based on ultrasound, magnetic stimulation and balloon catheter-based recordings. Diaphragm dysfunction with impaired voluntary activation can be present 1 year after severe COVID-19 ARDS and may relate to exertional dyspnea.

This prospective case–control study was registered under the trial registration number NCT04854863 April, 22 2021

**Supplementary Information:**

The online version contains supplementary material available at 10.1186/s12931-022-02100-y.

## Introduction

Up to 30% of coronavirus disease 2019 (COVID-19) survivors report dyspnea on exertion that could not be explained by routine clinical diagnostic measures and prevented most of them from returning to their original work and life [1–3].

Symptoms of (former) COVID-19 patients have not yet been assessed in the context of respiratory muscle function using gold standard techniques. This is relevant because COVID-19 and/or its treatment with invasive mechanical ventilation (IMV) might impact on respiratory muscle function [4]. Therefore, this study assessed inspiratory muscle dysfunction and its central voluntary activation at 12 months after COVID-19-related acute respiratory distress syndrome (ARDS).

## Materials and methods

The present prospective case–control study (ClinicalTrials.gov Identifier: NCT04854863) was conducted ethically in accordance with the World Medical Association Declaration of Helsinki and was approved by the local ethics committee (Ethikkommission an der medizinischen Fakultät der Rheinisch-Westfälischen Technischen Hochschule Aachen, CTCA-A-Nr. 20-515, AZ EK 443/20) and written informed consent was obtained in every subject.

**Fig. 1:**
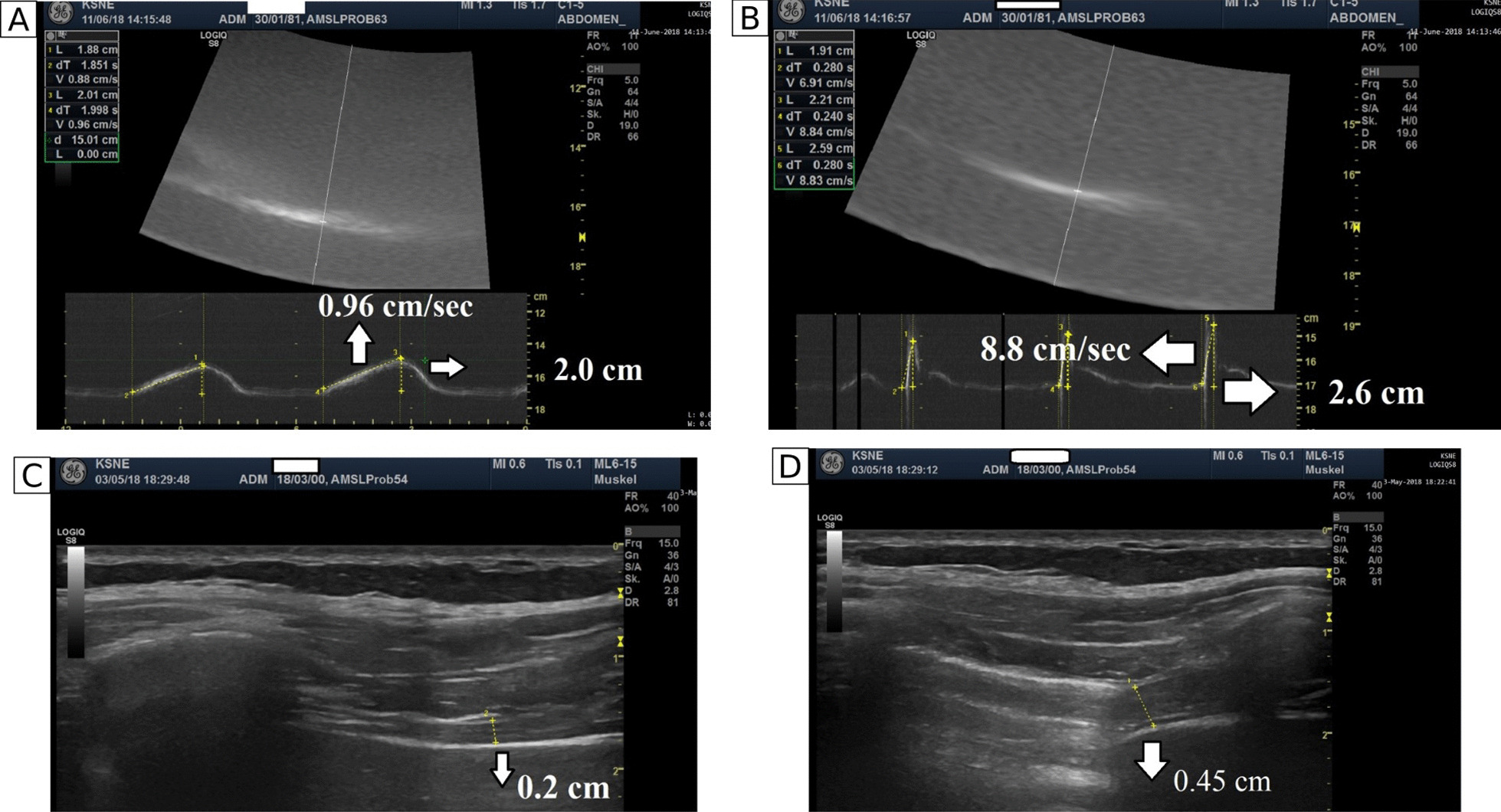
Parameters measured during diaphragm ultrasound: diaphragm excursion during tidal breathing (**A**) and sniff maneuver (**B**); and diaphragm thickness at functional residual capacity (FRC) (**C**) and at total lung capacity (TLC) (**D**)

Ten patients (6 female, age 56 ± 14 years) hospitalized for acute COVID-19 at the University Hospital RWTH Aachen in 2020 who were admitted to the intensive care unit (ICU) with ARDS requiring IMV for approximately 2 months (mean 63 ± 45 days) were evaluated at 1 year after discharge. The control group included healthy subjects propensity matched 1:1 for age, sex, and body mass index (BMI) [5–7]. All subjects underwent pulmonary function tests (PFTs), a 6-min walk test (6MWT), echocardiography (Fig. 1) [5], invasive recording of twitch transdiaphragmatic pressure (twPdi) following magnetic diaphragm stimulation, and diaphragm ultrasound (Fig. 1) [5–7]. Details on twPdi measurements, diaphragm ultrasound, determination of diaphragm voluntary activation index as well as the statistical analyses performed can be found in the Additional file 1.

## Results

All patients had severe COVID-19 with ARDS and were managed with IMV in the ICU. Two patients received extracorporeal membrane oxygenation therapy, seven developed acute renal failure requiring continuous renal replacement therapy, and eight needed prone positioning. Patients were discharged from hospital after a mean of ~ 2 months. None of the patients or the controls had been diagnosed with any comorbidity potentially impacting on diaphragm dysfunction (i.e. no systolic heart failure, no chronic obstructive pulmonary disease, no neuromuscular disorders). One year post discharge, none of the patients enrolled reported any further hospital admission for COVID-19-related medical issues.

Neither PFTs nor echocardiography showed significant abnormalities (Table 1). However, while four patients did not complain of relevant dyspnea (mild/no dyspnea [Borg dyspnea scale score of 0 or 1] following a 6MWT), six patients reported persisting dyspnea on exertion (severe in two [Borg dyspnea scale score ≥ 6], moderate in four [Borg dyspnea scale score 2–5]) despite normal lung function (FEV_1_ 96 ± 13% predicted, vital capacity 96 ± 10% predicted) and no abnormalities were seen on echocardiographic scans or comprehensive laboratory testing of blood samples (Table 1). More severe dyspnea on exertion was associated with shorter distances achieved on the 6MWT (554 ± 59 vs. 469 ± 54 vs. 316 ± 177 m across the three dyspnea subgroups, ANOVA p = 0.04) (Table 1). All patients complained of dyspnea on exertion but not at rest; none had experienced dyspnea before being ill with COVID-19.

**Table 1:** PFTs, 6MWT, echocardiography and laboratory findings at 12 months follow up and according to dyspnea on exertion

	COVID 19 patients (n = 10)	No/mild dyspnea (n = 4)	Moderate dyspnea (n = 4)	Severe dyspnea (n = 2)	p-value*
Pulmonary function and ABGs					
TLC, % of predicted	100.44 ± 10.83	101.58 ± 9.74	104.03 ± 13.28	91.00 ± 2.69	n.s
VC, % of predicted	96.15 ± 9.99	97.20 ± 9.08	100.08 ± 11.22	86.20 ± 2.97	n.s
RV, % of predicted	97.15 ± 42.72	114.25 ± 52.09	98.35 ± 22.51	60.55 ± 53.95	n.s
RV/TLC, % of predicted	105.64 ± 17.21	105.08 ± 17.31	104.43 ± 21.73	109.20 ± 17.82	n.s
FEV_1_, % of predicted	96.20 ± 13.08	98.95 ± 12.84	98.63 ± 15.99	85.85 ± 3.18	n.s
FEV_1_/FVC, %	79.98 ± 10.40	79.60 ± 5.70	79.71 ± 16.29	81.25 ± 8.75	n.s
Reff, % of predicted	91.41 ± 20.09	98.85 ± 12.77	87.98 ± 24.47	83.40 ± 30.83	n.s
DLCO/VA, % predicted	74.74 ± 18.31	86.00 ± 14.16	65.13 ± 19.68	68.30 ± 12.50	n.s
PaO_2_, mmHg	76.90 ± 16.08	66.88 ± 8.50	77.73 ± 9.11	75.25 ± 32.17	n.s
PaCO_2_, mmHg	35.15 ± 5.21	40.08 ± 3.93	32.85 ± 3.22	39.75 ± 6.43	n.s
pH	7.43 ± 0.07	7.41 ± 0.03	7.46 ± 0.07	7.38 ± 0.04	n.s
Base excess, mmol/l	− 0.52 ± 2.32	0.73 ± 1.07	0.23 ± 2.56	− 2.00 ± 0.99	n.s
6MWT					
Distance, m	471.90 ± 118.53	553.50 ± 58.95	468.75 ± 54.37	315 ± 176.78	**0.04**
SpO_2_ after exercise, %	94.67 ± 1.75	94.00 ± 0.82	94.00 ± 1.20	98.00 ± 1.89	n.s
Echocardiography					
LVEF > 50%, n (%)	10 (100)	4 (100)	4 (100)	2 (100)	n.s
LVEDD, mm	49.00 ± 2.34	44.25 ± 4.92	50.00 ± 2.94	49.00 ± 0.00	n.s
IVSD, mm	11.0 ± 1.79	10.25 ± 1.71	11.25 ± 2.22	10.50 ± 0.71	n.s
Left atrial area, cm^2^	20.20 ± 3.83	18.00 ± 3.56	19.67 ± 3.51	21.00 ± 5.66	n.s
TAPSE ≥ 18 mm, n (%)	10 (100)	4 (100)	4 (100)	2 (100)	n.s
Right atrial area, cm^2^	10 (100)	4 (100)	4 (100)	2 (100)	n.s
Hematology					
White blood cells, 1/nL	6.67 ± 1.13	6.03 ± 0.50	7.40 ± 1.41	6.50 ± 0.99	n.s
Hemoglobin, g/dL	14.47 ± 1.77	14.80 ± 1.21	14.87 ± 2.05	13.00 ± 2.40	n.s
Platelets, 1/nL	237.90 ± 53.71	233.75 ± 54.73	252.25 ± 58.73	217.50 ± 70.00	n.s
Lymphocytes, %	27.41 ± 9.67	31.40 ± 8.52	25.85 ± 12.61	22.55 ± 5.16	n.s
Coagulation					
D-dimer, ng/mL	486 ± 301	344 ± 273	541 ± 371	552.00 ± 295	n.s
Clinical chemistry					
LDH, U/L	184 ± 18	190 ± 32	179 ± 11	183 ± 11	n.s
CK, U/L	96.67 ± 41.40	122.00 ± 62.45	93.00 ± 24.25	66.00 ± 16.97	n.s
hs-Troponin T, pg/mL	13.75 ± 7.48	12.50 ± 6.36	11.50 ± 3.70	19.50 ± 14.85	n.s
Creatinine, mg/dL	1.11 ± 0.25	1.00 ± 0.18	1.12 ± 0.34	1.25 ± 0.21	n.s
CRP, mg/L	3.53 ± 5.38	0.90 ± 0.36	4.98 ± 7.44	4.60 ± 5.80	n.s
PCT, ng/mL	0.08 ± 0.14	0.01 ± 0.00	0.06 ± 0.04	0.24 ± 0.29	n.s
Cytokines					
IL-6, pg/mL	3.68 ± 3.46	1.53 ± 0.23	4.41 ± 4.37	5.46 ± 4.04	n.s

Bold indicates p value < 0.05

Values are mean ± standard deviation or number of patients (percentage). *ANOVA

*ABGs* arterial blood gases, *BP* blood pressure, *CK* creatine kinase, *CRP* C-reactive protein, *DLCO* diffusing capacity for carbon monoxide, *FEV*_*1*_ forced expiratory volume in 1 s, *FVC* forced vital capacity, *IVSD* inter-ventricular septal thickness in diastole, *LDH* lactate dehydrogenase, *LVEDD* left ventricular end-diastolic diameter, *LVEF* left ventricular ejection fraction, *6MWT* six-min walk test, *PaCO*_*2*_ partial pressure of carbon dioxide, *PaO*_*2*_ partial pressure of oxygen, *PCT* procalcitonin, *PFTs* pulmonary function tests, *Reff* effective specific resistance, *RV* residual volume, *SpO*_*2*_ oxygen saturation, *TAPSE* tricuspid annular plane systolic excursion, *TLC* total lung capacity, *VA* alveolar volume, *VC* vital capacity, *hs-Troponin-T* high sensitive troponin-T *IL-6* interleukin-6, *LDH* lactate dehydrogenase

On ultrasound, diaphragm function was clearly impaired with an abnormal diaphragm thickening ratio (2.76 ± 0.72 in post COVID-19 patients vs. 1.87 ± 0.37 in controls; p < 0.01) and diaphragm excursion velocity during a maximum sniff maneuver was associated with dyspnea on exertion (7.00 ± 0.82 vs. 6.95 ± 1.33 vs. 3.25 ± 1.77 cm/sec across the three dyspnea subgroups; ANOVA p = 0.02) (Table 2). This was supported by invasively obtained muscle pressure recordings (both Sniff PDI and Mueller PDI as volitional metrics reflecting inspiratory muscle strength were reduced across the three dyspnea subgroups) (Table 2).

**Table 2:** In-depth analysis of respiratory muscle function in post-COVID-19 acute respiratory distress syndrome (ARDS) patients versus control, and based on dyspnea on exertion presence/severity, at 1-year follow-up

	Controls (n = 10)	Patients with COVID-19 (n = 10)	p-value	Dyspnea level in patients with COVID-19			
				No/mild (n = 4)	Moderate (n = 4)	Severe (n = 2)	p-value*
Age (years)	61 ± 7	58 ± 9	n.s	–	–	–	n.s
Proportion of males, %	70	70	n.s	–	–	–	n.s
Non-volitional invasive RMS							
CMS TwPdi, cmH_2_O [LLN. 19.0 (M/F)]	22 ± 6	20 ± 8	n.s	16 ± 4	26 ± 10	17 ± 1	n.s
COMS TwPdi, cmH_2_O [LLN. 9.7 (M), 11.3 (F)]	14 ± 9	16 ± 9	n.s	21 ± 11	13 ± 4	11 ± 17	n.s
Volitional invasive RMS							
Sniff Pdi, cmH_2_O [LLN. 78 (M), 57 (F)]	79 ± 24	71 ± 30	n.s	92 ± 40	57 ± 8	57 ± 1	**0.04**
Sniff Pes, cmH_2_O [LLN. − 57 (M), − 41 (F)]	− 54 ± 16	− 54 ± 27	n.s	− 71 ± 38	− 46 ± 11	− 38 ± 8	n.s
Mueller Pdi, cmH_2_O [LLN. 63 (M), 48 (F)]	80 ± 38	52 ± 41	n.s	66 ± 26	57 ± 55	25 ± 14	**0.05**
Mueller Pes, cmH_2_O [LLN. − 11 (M), − 13 (F)]	− 26 ± 25	− 35 ± 38	n.s	− 40 ± 31	− 39 ± 34	− 20 ± 4	n.s
Twitch interpolation							
DVAI, % [LLN. 31 (M/F)]	73 ± 6	48 ± 17	**< 0.01**	62 ± 9	46 ± 8	23 ± 3	**0.02**
Diaphragm ultrasound							
Amplitude TB, cm [LLN. 1.2 (M/F)]	1.46 ± 0.61	1.46 ± 0.61	n.s	1.25 ± 0.29	1.60 ± 0.52	1.50 ± 0.71	n.s
Velocity TB, cm/sec [LLN. 0.8 (M/F)]	1.202 ± 0.59	1.12 ± 0.57	n.s	1.25 ± 0.50	1.20 ± 0.24	1.00 ± 0.73	n.s
Sniff velocity, cm/sec [LLN. 6.7 (M), 5.2 (F)]	6.22 ± 1.26	6.23 ± 1.90	n.s	7.00 ± 0.82	6.95 ± 1.33	3.25 ± 1.77	**0.02**
Thickness at FRC, cm [LLN. 0.17 (M), 0.15 (F)]	0.22 ± 0.12	0.21 ± 0.03	n.s	0.22.0.03	0.23 ± 0.05	0.21 ± 0.01	n.s
Thickness at TLC, cm [LLN 0.46 (M), 0.35 (F)]	0.58 ± 0.27	0.39 ± 0.08	**0.05**	0.39 ± 0.11	0.40 ± 0.08	0.39 ± 0.01	n.s
DTR [LLN 2.2 (M/F)]	2.76 ± 0.72	1.87 ± 0.37	**< 0.01**	1.76 ± 0.38	1.91 ± 0.48	1.86 ± 0.19	n.s
DTf, % [LLN 120 (M/F)]	126 ± 74	87 ± 37	**< 0.01**	76 ± 38	97 ± 48	86 ± 19	n.s

Bold indicates p value < 0.05

Values are presented as mean ± standard deviation or number of patients (percentage). *ANOVA. Lower limit of normal (LLN) values for males (M) and females (F) are fifth percentile values derived from previous studies by our group [5–7]

*CMS* cervical magnetic stimulation (of the phrenic nerve roots), *COMS* cortical magnetic stimulation (of the phrenic nerve roots), *DTR* diaphragm thickening ratio, *DTf* diaphragm thickening fraction, *DVAI* diaphragm voluntary activation index, *FRC* functional residual capacity, *PDI* transdiaphragmatic pressure, *Pes* esophageal pressure, *Pgas* gastric pressure, *RMS* respiratory muscle strength, *TB* tidal breathing, *TLC* total lung capacity, *TwPDI* twitch transdiaphragmatic pressure

**Fig. 2:**
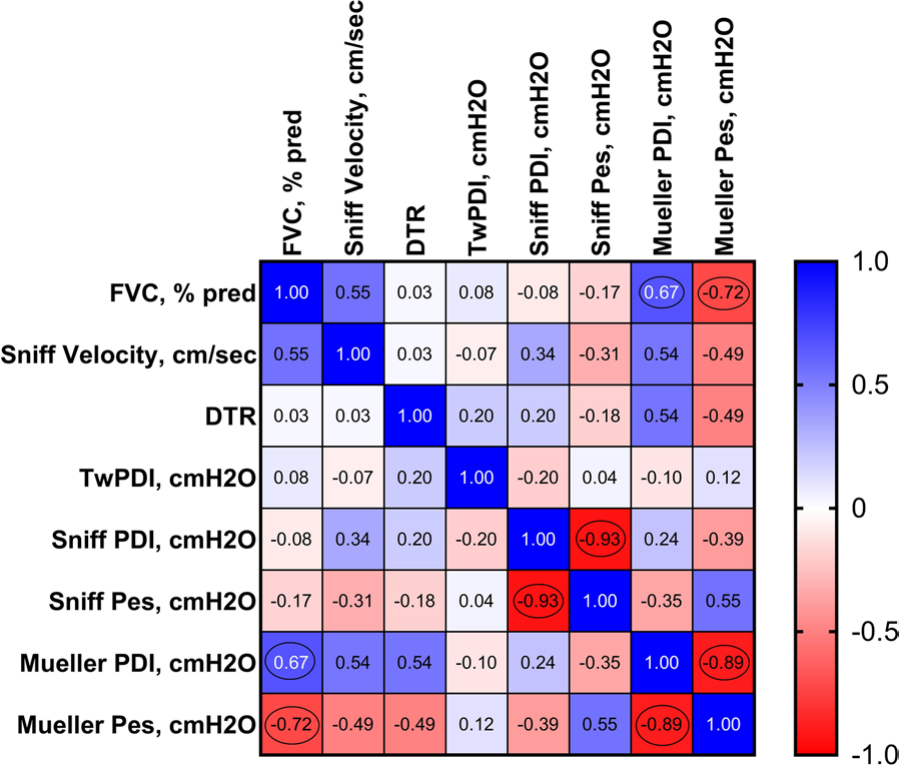
Associations between pulmonary function testing (forced vital capacity), twitch pressure (twPdi) plus volitional invasively obtained inspiratory pressure gradients (Mueller and Sniff maneuver) and diaphragm ultrasound data (DTR and Sniff velocity). Strength of correlation: weak (r = 0.20–0.39), moderate (r = 0.40–0.59), strong (r = 0.60–0.79) or very strong (r = 0.80–1.00); r-values with a corresponding p-value < 0.05 are circled. *DTR* diaphragm thickening ratio, *FVC* forced vital capacity, *PDI* diaphragmatic pressure, *Pes* esophageal pressure, *twPDI* twitch diaphragmatic pressure

However, twPdi following CMS did not differ between patients and controls overall (22 ± 6 20 ± 8 cmH_2_O, p = n.s.) (Table 2). Supramaximality of CMS was seen in all subjects based on a < 10% increase in twPdi amplitude when going from 80 to 90% (or even from 90 to 100%) power output of the magnetic coil. DVAI was lower in patients versus controls (73 ± 6 vs. 48 ± 17%; p < 0.01) (Table 2). The central reduction in diaphragm activation was associated with dyspnea on exertion (diaphragm voluntary activation index, 62 ± 9 vs. 46 ± 8 vs. 23 ± 3% across the three dyspnea subgroups; ANOVA p = 0.02) (Table 2). There were no other differences across the dyspnea subgroups in COVID-19 survivors, except for a longer duration of IMV in patients with dyspnea (Table 2). Only moderate-weak correlations (only very few of which achieved statistical significance were detected between PFT, DUS metrics and invasively measured actual strength values (Fig. 2).

## Discussion

This is the first study to show the presence of diaphragm dysfunction in post COVID-19 patients with ARDS, as determined using gold standard techniques. Further, the present study relates diaphragm dysfunction and its neural control to dyspnea on exertion 1 year after COVID-19 ARDS. Given that routine work-up did not reveal relevant impairment, our study suggests that diaphragm dysfunction may be a pathophysiological correlate of dyspnea on exertion in post COVID-19 patients. This is supported by the fact that diaphragm pathology has been reported in postmortem findings of patients who had been critically ill with COVID-19 [8].

It is not surprising to see that standard PFTs do not detect these changes in the respiratory musculature. Polkey and colleagues have previously demonstrated that in-depth respiratory musculature assessment techniques increase the accuracy of diaphragm dysfunction diagnosis by up to 40%. [9]

Our data may also indicate that volitional (DTR, sniff velocity, pressures achieved in sniff manoeuvre) rather than non-volitional (twPdi curves following CMS) metrics of inspiratory muscle function are impaired in post COVID-19 patients and relate to the sensation of dyspnea on exertion. This points towards the theory that central “neural” control of the diaphragm rather than “peripheral contractility” underly diaphragm dysfunction. The present study also directly showed that there is a central, “neural” contribution to diaphragm dysfunction in COVID-19 ARDS survivors by demonstrating that the DVAI was significantly lower in these patients. While previous research in this area is scarce, clincally it appears plausible to link impaired volitional metrics of diaphragm function and its neural control to the sensation of dyspnea on exertion. This is because such impairements reflect the inabilty of the respiratory muscle pump to maintain sufficient ventilation on exertion, the mismatch of which may be perceived as dyspnea by the patient.

From a methodological point of view, the present work makes a contribution to the relationship between diaphragm ultrasound-derived metrics and invasively obtained actual strength values. Only moderate-weak correlations were documented between PFT, diaphragm ultrasound metrics and invasively-measured strength values. This is consistent with previous work from our group and shows that ultrasound only provides surrogate markers of diaphragm function without reflecting its actual strength. This is probably because a three-dimensional pressure-generating process is captured in a two-dimensional ultrasound picture, and only one (standardized) part of the diaphragm is assessed to determine velocity and contraction capacity [10]. Therefore, clinically, diaphragm ultrasound supplements, but does not replace, invasive measurements when diagnosing diaphragm muscle weakness.

While the number of patients recruited was quite small, our data are hypothesis generating and can inform design future studies with more patients, including those not managed using IMV, to investigate whether IMV (through a loss in respiratory muscle mass [11]) or SARS-CoV-2 infection per se (potentially through its affinity to neural tissue [1–3]) is causing diaphragmatic dysfunction. Yet, the small sample size must also be kept in mind for -potentially- not reaching statistical significant results also with regard to the correlation coefficients calculated. Predisposition to developing diaphragm dysfunction in long-term ventilated patients was documented prior to the COVID-19 pandemic, especially in the presence of major ARDS with lung lesions that could persist years later [12, 13]. Severe COVID-19 often necessitates a long period of IMV and it is also possible that SARS-CoV-2 infection itself could cause diaphragmatic dysfunction, both of which could potentially contribute to significant impairment of diaphram dysfunction over the long term, and this could be related to persistent dyspnea, as reported for the first time in our patients.

In conclusion, inspiratory muscle dysfunction, with impaired central activation of the diaphragm in particular, is present 1 year after IMV for COVID-19-related ARDS, and this may relate to dyspnea on exertion.

## Supplementary Information

**Additional file 1.** Online Supplemental material: Materials and Methods.

## Data Availability

All data can be made available upon reasonable request.
