# Supplementary material for: Diaphragm dysfunction as a potential determinant of dyspnea on exertion in patients 1 year after COVID-19-related ARDS
Source: Respir Res. 2022 Jul 15;23:187. doi: 10.1186/s12931-022-02100-y (PMC9284093; doi:10.1186/s12931-022-02100-y)
Supplement: Supplementary file 1 — Additional file 1. Online Supplemental material: Materials and Methods. [file 12931_2022_2100_MOESM1_ESM.docx]

**Online Supplemental material: Materials and Methods**

**Diaphragm dysfunction as a potential determinant of dyspnea on exertion in patients** **one year after COVID-19-related ARDS**

Jens Spiesshoefer^1,2*^, Janina Friedrich^1*^, Binaya Regmi^1^, Jonathan Geppert^1^, Benedikt Jörn^1^, Alexander Kersten^3^, Alberto Giannoni^2^, Matthias Boentert^4,5^, Gernot Marx^6^, Nikolaus Marx^3^, Ayham Daher^1^, Michael Dreher^1^

^1^Department of Pneumology and Intensive Care Medicine, University Hospital RWTH Aachen, Aachen, Germany; ^2^Institute of Life Sciences, Scuola Superiore Sant’Anna, Pisa, Italy; ^3^Department of Cardiology, Vascular and Intensive Care Medicine, University Hospital RWTH Aachen, Aachen, Germany; ^4^Department of Neurology with Institute for Translational Neurology, University Hospital of Muenster, Muenster, Germany; ^5^Department of Medicine, UKM Marienhospital Steinfurt, Steinfurt, Germany; ^6^Department of Intensive Care and Intermediate Care, University Hospital Rheinisch Westfaelische Technische Hochschule Aachen, Aachen, Germany

^*^Jens Spiesshoefer and Janina Friedrich contributed equally to this work.

**Running head:** Diaphragm dysfunction and dyspnea on exertion after COVID-19

**Corresponding author:** Dr. Jens Spiesshoefer, Department of Pneumology and Intensive Care Medicine, University Hospital RWTH Aachen, Aachen, Germany; E-Mail: [jspiesshoefer@ukaachen.de](mailto:jspiesshoefer@ukaachen.de)

**Online Supplemental material: Materials and Methods**

Phrenic nerve conduction studies were performed as previously described.^6^ Cortical magnetic stimulation (COMS) and posterior cervical magnetic stimulation (CMS) were performed with the subject in a seated position. Stimuli were delivered using a MagPro Compact^TM^ magnetic stimulator equipped with a 2 Tesla 12 cm C-100 circular coil (MagVenture, Willich, Germany). For COMS the coil was positioned over Cz´ according to the international 10-20 electroencephalograph system.^6^ For posterior CMS the coil was placed at C7 and then moved up towards C6 until the highest reproducible twitch transdiaphragmatic pressure (twPDI) was obtained.^6^ At least five stimuli were delivered to achieve the highest possible twPDI (both for COMS and posterior CMS) showing <10% variation from the preceding two stimulations.^6^ Supramaximality of magnetic stimuli (with 0.1 msec duration each and 2.0 Tesla maximum magnetic field output) was achieved judging the relationship between stimulation intensity and amplitude of the twPDI. Stimuli were separated by ≥30 seconds to avoid twitch potentiation.^6^ Stimulation at functional residual capacity (FRC) was determined by visual observation of abdominal movements.

Twitch esophageal pressure (twPes) and twitch gastric pressure (twPgas) were simultaneously recorded using balloon catheters (Cooper Surgical, Trumbull, CT, USA) transnasally inserted into the stomach and the distal esophagus as previously described.^6^ Balloon catheters were connected to a differential pressure transducer (DPT-100TM, Utah Medical Products, Athlone, Ireland) and a carrier amplifier (ADInstruments, Oxford, UK).^6^ Pressure data for twPgas, twPes and twPdi (defined as twPes–twPgas) were continuously displayed using LabChartTM software (ADInstruments, Oxford, UK).^6^

The diaphragm voluntary activation index (DVAI) was determined as a measure of central respiratory drive; it reflects the percentage of diaphragmatic muscle mass activated by voluntary effort or the extent of diaphragmatic activation during any given inspiratory effort.^7^ Therefore, subjects were instructed to perform a maximum inspiratory effort against an occluded airway at FRC. DVAI was calculated as:

$$DVAI=1-\frac{twPdi at max. inspiratory effort (i.e. diaphragm voluntarly contracted)}{twPdimax at FRC (i.e. diaphragm relaxed)} x 100$$

A portable ultrasound machine (LOGIQ S8 -XD clear, GE Healthcare, London, United Kingdom) with a 3.5 MHz convex transducer was used to assess diaphragm excursions in the subcostal view, and a 10 MHz linear transducer was used for evaluation of diaphragm thickness in the zone of apposition. Measurements were performed on the right hemidiaphragm in the supine position because posture is known to directly affect diaphragm thickness. All sonographic recordings were saved for later analysis. All measurements were performed three times and the average value for each parameter was calculated. For evaluation of diaphragm excursions, the 3.5 MHz probe was positioned between the mid-clavicular and anterior axillary lines, in the subcostal area with the probe held as medially as possible and directed cranially.^5^ Excursions of the right hemidiaphragm were recorded on M-mode sonography in real time with a clear instruction to “cut” the hemidiaphragm in its posterior third (Figure 1). Measurement of diaphragm excursion amplitude was performed during tidal breathing (TB) and following a voluntary sniff (VS), which leads to maximum displacement of the diaphragm (Figure 1).^5^ Assessment of diaphragm excursion velocity was performed during TB and following the VS maneuver only (Figure 1).^5^ Excursion amplitude was defined as the upright-perpendicular distance from the minimum to the maximum point of diaphragm displacement, and excursion velocity was defined as the upright-diagonal distance between these points (Figure 1).

Diaphragm thickness was measured as the vertical distance between the pleural and peritoneal layer at both total lung capacity (TLC) and functional residual capacity (FRC) (Figure 1).^5^ This was done in the zone of apposition with the 10 MHz probe positioned in the posterior axillary line between the eighth and tenth intercostal space (Figure 1).^5^ Diaphragm thickening ratio (DTR) was calculated as thickness at TLC divided by thickness at FRC (Figure 1).^5^

This was also expressed by calculating and providing the diaphragm thickening fraction (DTf; %) that was calculated as the difference between diaphragm thickness at TLC and at FRC divided by diaphragm thickness at FRC ×100.

Statistical analyses were performed using Sigma Plot^TM^ software (Version 13.0, Systat, Erkrath, Germany). The primary endpoint was a reduction in DTR. Assuming a two-sided significance level of 0.05 (alpha) and 80% power (beta), a sample size of nine subjects per group was calculated to allow detection of a 25% difference in twPdi.^5,6,7^ Normal values for DTR in healthy individuals were derived from our previous work.^5^

Data are expressed as mean ± standard deviation, and the t-test for independent samples was used for group comparisons. Differences between the three patient groups (no/mild, moderate and severe dyspnea) were tested using one-way ANOVA with Tukey post-hoc test for pairwise comparisons when normal distribution could be assumed. Otherwise, the Kruskal-Wallis test with Bonferroni post-hoc tests was used. For comparisons between two groups, Fisher’s exact t-test or the Mann-Whitney U test was used as appropriate.

Pearson product moment correlation was used for simple linear regression analysis to explore associations between PFT (forced vital capacity), twitch pressures (twPdi) plus volitional invasively obtained inspiratory pressure gradients (Mueller and Sniff maneuver) and data derived from diaphragm ultrasound (DTR and Sniff velocity in particular). Strength of correlation was classified as weak (0.20–0.39), moderate (0.40–0.59), strong (0.60–0.79) or very strong (0.80–1.00). A p-value of ≤0.05 was considered statistically significant.
